# Effects of Copper Pollution on the Phenolic Compound Content, Color, and Antioxidant Activity of Wine

**DOI:** 10.3390/molecules22050726

**Published:** 2017-05-03

**Authors:** Xiangyu Sun, Tingting Ma, Luyang Han, Weidong Huang, Jicheng Zhan

**Affiliations:** College of Food Science and Nutritional Engineering, Beijing Key Laboratory of Viticulture and Enology, China Agricultural University, Beijing 100083, China; sunyu7459@cau.edu.cn (X.S.); matingtingwz@163.com (T.M.); hanluyang001@126.com (L.H.); huanggwd@263.net (W.H.)

**Keywords:** copper, wine, phenolic compounds, color, antioxidant activity, HPLC, CIELAB

## Abstract

The effects of copper pollution on the polyphenol content, color, and antioxidant activity of wine, as well as correlations among these factors, were investigated. Copper had clear influences on wine polyphenol content. At low copper concentrations, the concentrations of nearly all polyphenols increased, and the antioxidant activity values of the wine also increased. When the copper concentration reached the lowest level of the medium copper range (9.6~16 mg/L), most of the indices also improved. When the copper concentrations reached the latter part of the medium copper range (19.2 and 22.4 mg/L), many of the tested indices began to decrease. Furthermore, when the copper concentration reached the high ranges (32, 64, and 96 mg/L), the polyphenol content, CIELAB color parameters, and antioxidant activity of wine were substantially decreased, indicating the need to control increasing copper content in grape must.

## 1. Introduction

Copper (Cu) is one of the heavy metals of greatest concern in the wine industry [1,2,3,4,5]. Long-term use of copper fungicides (mainly the Bordeaux mixture), which increases the copper levels in soil and grape berries [5,6,7], and the use of copper winemaking equipment [8], and copper sulfate or copper citrate additives to eliminate H_2_S [9,10] may also increase the copper contents in grape must and wine. Of these sources, copper fungicides, which have been used in unlimited high doses in vineyards for many decades (since the 1880s in France) [3], are of specific concern. These fungicides not only leave residues on grape skin that are directly transmitted into grape must and wine, but also accumulate in vineyard soils, as copper rarely degrades or moves in arable soil layers, which causes vineyard soils to become copper-enriched [5]. The soil copper content in some vineyards has increased far beyond the European Union (EU) regulatory limit of 140 mg/kg, in some cases reaching 1500 mg/kg [6,9,11]. Very large amounts of copper are transported to the grapes from the roots through the transpiration system and can affect the quality of the grapes, grape must, and wines [11]. In fact, copper concentrations in grape must increase with the number of copper fungicide applications [3].

In the International Organization of Vine and Wine (OIV), the EU and South Africa (S.A.), the maximum tolerance limits for copper residue are 20 mg/L and 1 mg/L in grape must and in wines, respectively [9,12]. In previous reports, the range of copper contents in grape must and wines varied greatly [9,12,13], and copper contents exceeding these limits are occasionally detected. Based on a report from Italy, approximately 13% of grapes and 18% of wines exceeded the maximum copper residue limits [9]. Copper content has also exceeded the maximum limits in Piedmont (Italy), Australia, Jordan, and China [13,14,15,16]. The Bordeaux mixture pesticide will be difficult to replace in the near future, and the copper contents of vineyard soils and grapes will continue to increase. In the future, we will probably need to contend with wine fermentation in the presence of high copper concentrations and the concomitant problem of reducing the copper concentration in wines [4].

Wine is a complex matrix that contains many classes of compounds [17], of which phenolic compounds are related to several of the main characteristics of wine [18]. These compounds have been shown to significantly influence the sensory characteristics of wines, such as mouthfeel, color, palatability, bitterness, and astringency. Similar to other foods, these compounds are the main contributors to the antioxidant capacity of wine [18,19]. These constituents are divided into the following two groups based on their chemical formulae: flavonoid compounds and non-flavonoid compounds [18]. Many factors influence the phenolic compound contents in wines, such as sulfur dioxide (SO_2_), grape variety, vineyard soil, geographical location, and climate conditions (referred to as “terroir”), vinification technology, grape qualities, such as pesticide residues and metal contents in grape must, and the aging process, including in-barrel and in-bottle, etc. [17,18,20,21,22]. SO_2_, especially, plays the pivotal role on antioxidant capacity in commercial wine production [23,24].

At low concentrations, copper is an essential trace element that plays important positive roles in nearly all organisms [12]. However, when the concentrations exceed the beneficial range, copper can have an inhibiting effect, and even toxic effects, on cells [22]. In wine-making, a high copper content in grape must also affects the wine fermentation process and quality. When the copper concentration is greater than 20 mg/L, the growth of *Saccharomyces cerevisiae* (*S. cerevisiae*) is inhibited, resulting in delayed fermentation and reduced alcohol production. Furthermore, a series of reactions that influence the quality of wines, such as copper browning and wine oxidative browning, can occur [12,22]. These reactions specifically affect volatile sulfur compound concentrations, protein instability, and pigment deposition [12,23,24,25]. However, although a few reports have been published in wine or model wine systems [26,27], researchers have not clearly determined whether copper influences wine polyphenol content, requiring further systematic research.

Hence, in this paper, we studied the effects of different concentrations of copper pollution in grape must (low, medium, and high) on the phenolic compound contents to simulate the different levels of copper pollution observed during wine production. Furthermore, the color parameters (measured using the CIELAB method) and the antioxidant capacity of wines, which are significantly influenced by phenolic compounds, were also investigated. Based on these results, we will obtain a better understanding of the influence of copper pollution on wine production, thereby providing a potential strategy for improvement to the wine industry.

## 2. Results and Discussion

### 2.1. Changes in the Total Phenolic (TP), Total Flavonoid (TFA), Total Flavan-3-ol (TFO), and Total Anthocyanin (TA) Contents in Wine with Different Copper Concentrations

Three different copper content ranges, including low copper contents, 0 mM (also as a control group) and 0.05 mM (3.2 mg/L); medium copper contents, 0.15 mM (9.6 mg/L), 0.20 mM (12.8 mg/L), 0.25 mM (16.0 mg/L), 0.30 mM (19.2 mg/L, on the verge of the maximum limits), and 0.35 mM (22.4 mg/L, slightly above the maximum limits); and high copper contents, 0.5 mM (32 mg/L), 1.0 mM (64 mg/L), and 1.5 mM (96 mg/L) were used to analyze the effects of copper pollution on the phenolic compound content, color, and antioxidant activity of wine based on the maximum tolerance limit for copper residue in the OIV and EU (20 mg/L) and the actual copper content in grape must observed during wine production [2,4,5,9,12,13,14,15,16]. Different levels of copper pollution observed during wine production and representing both current and future values were simulated using this graded copper series.

**Figure 1:**
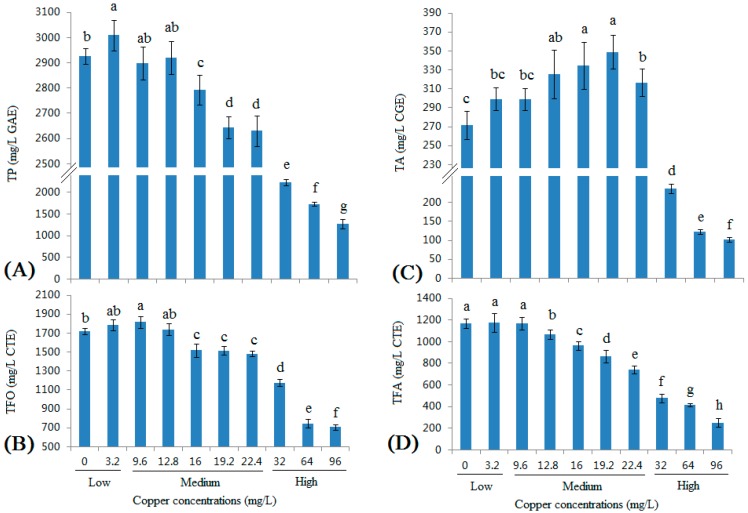
Changes in the TP (Total polyphenol) (**A**), TFO (total flavan-3-ol) (**B**), TFA (total flavonoid) (**C**), and TA (total anthocyanin) (**D**) contents in wine with different copper concentrations. Different letters indicate statistically significant differences (Duncan’s multiple range test, *p* < 0.05) between samples with different copper concentrations.

The TP, TFO, TFA, and TA contents in wine were measured at different copper concentrations are shown in Figure 1. The TP content significantly increased in the 3.2 mg/L group (Figure 1A) and remained stable in the 9.6 and 12.8 mg/L groups; in the 16–22.4 mg/L group, the TP content was slightly decreased. When the copper concentration reached the high ranges (32–96 mg/L) in the grape must, which were many times greater than the maximum limit in the grape must (20 mg/L), the TP content was substantially decreased, particularly in the 96 mg/L group, in which the TP content was only 1270.48 ± 106.01 mg/L, and only half of the value of the control group (Cu at 0 mg/L).

The TFO contents showed a similar trend as the TP contents (Figure 1B). In contrast to the TP contents, the highest TFO content was observed in the 9.6 mg/L group, whereas the 16, 19.2, and 22.4 mg/L groups showed similar contents. In the 32 mg/L group, the TFO contents were significantly decreased, and the TFO contents in the 64 and 96 mg/L groups were only one-third of the concentration in the 0 mg/L group. Moreover, these two groups showed similar contents, indicating that the effect of copper on the TFO contents was saturated at certain copper concentrations (64 mg/L).

Copper increased the TA contents (Figure 1C) in a dose-dependent manner to levels near the legal limit of copper, with the highest values detected in the 16 and 19.2 mg/L groups. When the copper concentration reached the high range (32–96 mg/L), the TA content was also substantially decreased, similar to the TP and TFO contents.

Low concentrations of copper had no effect on the TFA contents (Figure 1D), but the TFA content was substantially decreased as the copper concentration increased. In fact, copper showed the strongest negative effect on the TFA contents; even when the copper concentrations were within the legal limit (12.8 mg/L, 16 mg/L, and 19.2 mg/L), the TFA concentrations were substantially decreased (8.69%, 17.55%, and 26.19%, respectively). When the copper concentration exceeded the legal limit (22.4 mg/L, 32 mg/L, 64 mg/L, and 96 mg/L), the percent decreases were even greater (36.68%, 59.16%, 64.36%, and 78.55%, respectively).

Linear correlation coefficients were used to more intuitively observe the relationship between copper concentrations and the phenolic compound content of wine (Table 1). The contents of all types of phenolic compounds were positively correlated with the low copper concentrations. However, the contents of all phenolic compounds were negatively correlated with the medium copper concentration, with the exception of the TA contents. Moreover, the TP, TFO, TA, and TFA contents all showed negative correlations with the high copper concentration.

To summarize the present findings of Figure 1 and Table 1, it could be seen that low concentration copper clearly increased all kinds of phenolic compounds except TFA, but the reason remains unclear. When the copper concentration continues to increase to medium copper ranges of copper concentrations, the influence began to change. TP, TFO, and TFA began decreasing at 12.8 or 16 mg/L, this might be because, firstly, polyphenols could undergo complexation reactions with copper [27,28]. Secondly, copper could enhance the rate of oxidation of flavanols. Under these effects, the TP, TFO, and TFA decreased within the medium copper ranges. From this, it could be seen that, in fact, TFA might also been extracted more at low concentrations, but decreased by the complexation reaction, hence, appearing as having no change. However, TA continued increasing in medium copper ranges, which might be due to copper mediating the production of phenolic pigment [23,24]. At high copper concentration, all kinds of phenolic compounds dropped greatly, especially at 64 and 96 mg/L. Except the complexation reaction and oxidation reaction, there might be some other reasons. Firstly, high copper concentration could obviously inhibit the fermentation performance of *S. cerevisiae*, especially alcoholic fermentation, which would obviously affect the extraction of polyphenols from grape must [5]. Secondly, high copper concentration would significantly decrease the cell growth and viability of *S. cerevisiae* [4,29], which would also inhibit the extraction of polyphenols from grape must. Thirdly, after fermentation, in high copper concentration groups, the copper contents which were left in finished wine were much higher than in low and medium copper concentration groups (Table 2). In fact, in all three high copper concentration groups, the residual copper was many times higher than the standards of OIV and EU in wines (1 mg/L) [4,5]. Such high levels in wines would also cause the decrease of phenolic compounds in wines. Fourth, as Figure S1 shows, although in all 10 tested groups, the fermentation could finish, there still existed significant difference among the 10 groups. In the high copper concentration groups, though the OD_600_ value did not show great disparity (Figure S1A), the viable cell number (Figure S1B) and viable cell rate (Figure S1C) show significant differences among the low and medium copper concentration groups, which were all significantly lower than the viable cell number and viable cell rate in low and medium copper concentration groups, and caused a very low alcohol production (Figure S1D), which was in accordance with previous report [5,12]. The low viable cell number, the viable cell rate of *S*. *cerevisiae*, and the low alcohol concentration all would decrease the extraction of phenolic compounds from grape skins and seeds, resulting in the decrease of phenolic compounds in wines.

### 2.2. Changes in the Monomeric Phenolic Acid Contents in Wine with Different Copper Concentrations

**Figure 2:**
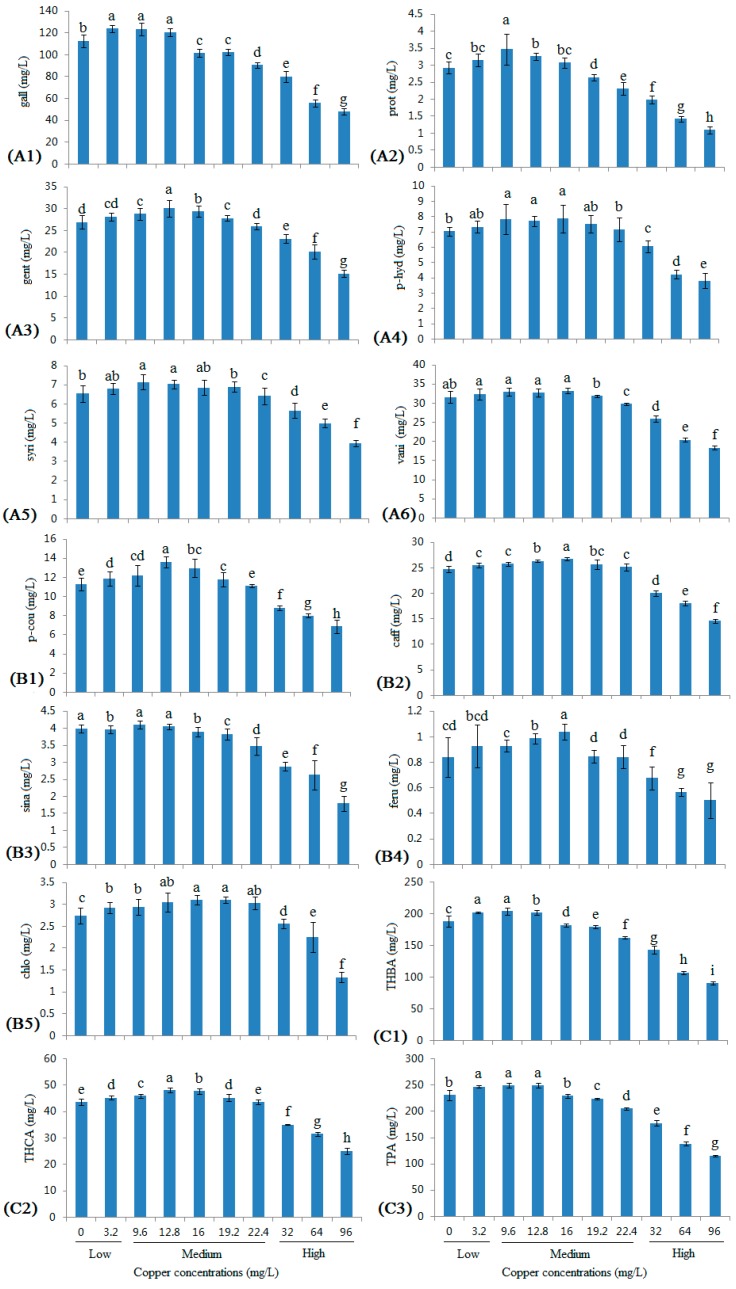
Changes in monomeric phenolic acid contents in wine with different copper concentrations. (**A1**) Gallic acid; (**A2**) protocatechuic acid; (**A3**) gentisic acid; (**A4**) *p-*hydroxybenzoic acid; (**A5**) syringic acid; (**A6**) vanillic acid; (**B1**) *p-*coumaric acid; (**B2**) caffeic acid; (**B3**) sinapic acid; (**B4**) ferulic acid; (**B5**) chlorogenic acid; (**C1**) total hydroxybenzoic acids; (**C2**) total hydroxycinnamic acids; and (**C3**) total phenolic acids. Different letters indicate statistically significant differences (Duncan’s multiple range test, *p* < 0.05) between samples with different copper concentrations.

Eleven monomeric phenolic acids and the total phenolic acids (TPA) were detected in this study (Figure 2), including six hydroxybenzoic acids and five hydroxycinnamic acids.

Of the hydroxybenzoic acids (Figure 2A1–A6), gallic acid was the major acid detected, similar to a previous report [18], followed by vanillic acid and gentisic acid. The levels of all six hydroxybenzoic acids increased as the copper concentration increased to 9.6 mg/L, with the exception of vanillic acid, which did not show significant changes at low copper concentrations. At medium copper ranges, the levels of gallic acid and protocatechuic acid showed a significantly decreasing trend, whereas only slight changes were observed in the levels of vanillic acid, *p-*hydroxybenzoic acid, gentisic acid, and syringic acid. At high copper ranges, the levels of all six hydroxybenzoic acids were substantially decreased, and the contents of the six hydroxybenzoic acids in the 96 mg/L group were approximately half that of the levels in the control group.

Caffeic acid and *p*-coumaric acid were the major hydroxycinnamic acids detected (Figure 2B1–B5), similar to a previous report [18]. Similar to the hydroxybenzoic acids, the contents of all five hydroxycinnamic acids showed a similar trend: a slight increase in samples with low copper concentrations and the lower range of the medium copper concentrations, and then a decrease over the upper range of the medium concentrations and high copper concentrations. Specifically, the levels of *p-*coumaric acid, ferulic acid, and chlorogenic acid showed a greater decrease.

The trends in the changing levels of the total hydroxybenzoic acids (THBA), the total hydroxycinnamic acids (THCA), and the TPA (Figure 2C1–C3) were also similar to the six hydroxybenzoic acids and five hydroxycinnamic acids. Compared with the control group, the THBA, THCA, and TPA levels retained 86.32%, 99.99%, and 88.90% of their initial values in the 22.4 mg/L group and retained 48.19%, 57.52%, and 49.95% of their initial values in the 96 mg/L group.

Based on the correlation coefficients (Table 1), the levels of all 11 phenolic acids were positively correlated with the low copper concentrations, with the exception of sinapic acid. The levels of *p-*hydroxybenzoic acid, caffeic acid, *p-*coumaric acid, ferulic acid, and chlorogenic acid were positively correlated, and the levels of the other six phenolic acids were negatively correlated with medium copper concentrations. The levels of all 11 phenolic acids and THBA, THCA, and TPA showed extremely significant negative correlations with the high copper concentrations. The same pathways may be responsible for the similar trends of the changes in the monomeric phenolic acid levels and the TP levels [5,23,27,28,29].

### 2.3. Changes in the Monomeric Flavan-3-ol Contents in Wine with Different Copper Concentrations

**Figure 3:**
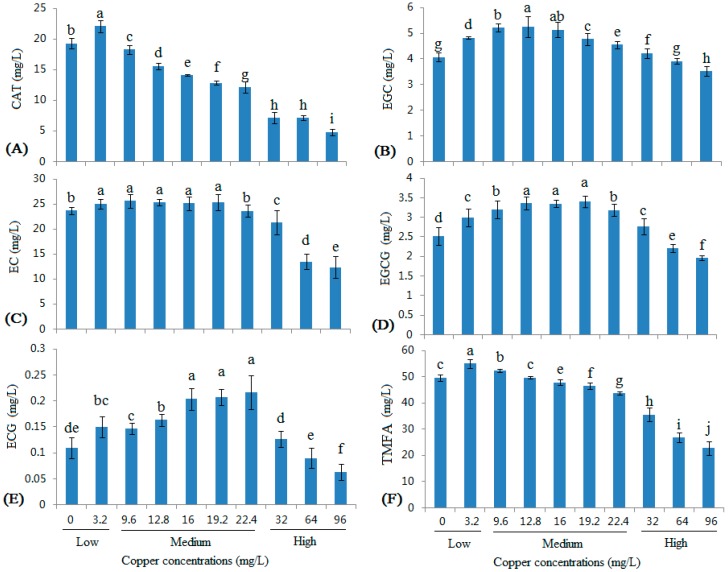
Changes in monomeric flavan-3-ol contents in wine with different copper concentrations. (**A**) CAT ((+)-catechin); (**B**) EC ((−)-epicatechin); (**C**) EGC ((−)-epigallocatechin); (**D**) ECG ((−)-epicatechin gallate); (**E**) EGCG ((−)-epigallocatechin gallate); and (**F**) total flavan-3-ols. Different letters indicate statistically significant differences (Duncan’s multiple range test, *p* < 0.05) between samples with different copper concentrations.

As shown in Figure 3, five monomeric flavan-3-ols were detected. The contents of all five flavan-3-ols increased at a copper concentration of 3.2 mg/L. At the medium copper range, the five flavan-3-ols showed different trends. The (+)-catechin (CAT) levels decreased at copper concentrations greater than 9.6 mg/L in a dose-dependent manner (Figure 3A). At copper concentrations greater than 9.6 mg/L, the (−)-epigallocatechin (EGC) contents significantly increased as the copper concentration increased, particularly in the 9.6 and 12.8 mg/L groups (Figure 3B); except that, in the 19.2 and 22.4 mg/L groups, it could be observed that the (−)-epicatechin gallate (ECG) was significantly increased (Figure 3E). Meanwhile, the (−)-epicatechin (EC) content did not change significantly (Figure 3C). When the copper pollution reached the high copper range, the levels of all five monomeric flavan-3-ols and the total monomeric flavan-3-ol acids (TMFA) were substantially decreased, similar to the changes in the TFA levels (Figure 1D).

Based on the correlation coefficients (Table 1), the levels of all five monomeric flavan-3-ols were positively correlated with low copper concentrations, similar to the monomeric phenolic acids. The EGC, EGCG, and ECG contents were positively correlated and the CAT and EC contents were negatively correlated with medium copper concentrations. The contents of all five flavan-3-ols and TMFA showed extremely significant negative correlations with high copper concentrations.

### 2.4. Changes in the CIELAB Color Parameters of Wine with Different Copper Concentrations

According to the OIV recommendation, wine color is measured using the L*, a*, and b* values, which are defined by the Commission Internationale Ed I'eclairage (CIE); this measurement has been called the CIELAB method [30]. Although this method cannot present an accurate definition of color, it effectively tracks changes in wine color during wine fermentation, aging, and storing. It achieves an objective evaluation of both wine color and any color changes using three specific quality attributes determined by visual perception: tonality, luminosity, and chroma [31,32]. The three-dimensional space of CIELAB includes luminosity (L*; L* = 0 represents black, and L* = 100 represents colorless), the red/green color component (a*; a* > 0 is associated with red, and a* < 0 is associated with green), and the yellow/blue color component (b*; b* > 0 is associated with yellow, and b* < 0 is associated with blue), from which the parameters correlated with color perception are obtained, including chroma (C) and hue angle (H) [30,31].

**Figure 4:**
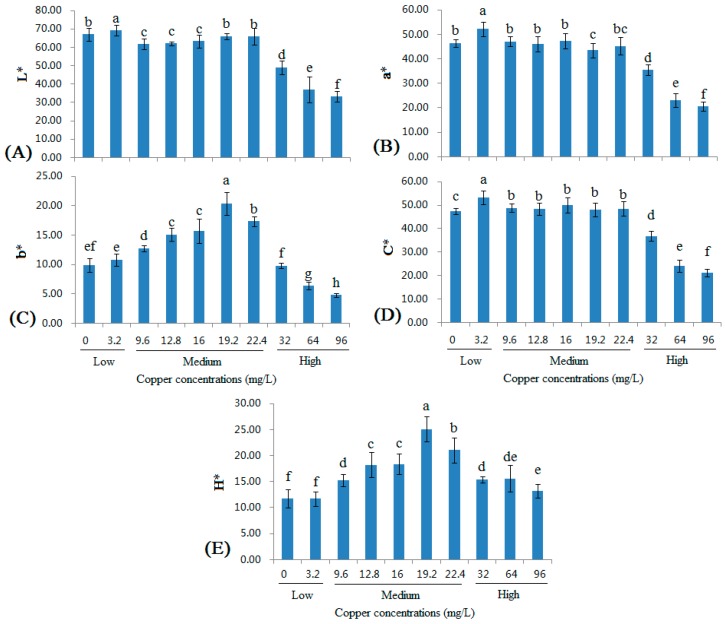
Changes in the L* (**A**), a* (**B**), b* (**C**), C* (**D**) and H* (**E**) values of wine with different copper concentrations. Different letters indicate statistically significant differences (Duncan’s multiple range test, *p* < 0.05) between samples with different copper concentrations.

As shown in Figure 4A, the changes in L* observed in wines with low and medium copper concentrations were not particularly large (although significant differences were observed). The L* value was substantially decreased in wines with high copper concentrations. The a* value increased in the 3.2 mg/L group, and then decreased and remained nearly stable in the 22.4 mg/L group. The a* value decreased, similar to the L* value in wines with high copper concentrations (Figure 4B). Compared with the L* and a* values, the trend for the changes in the b* value was much greater. As the copper concentration increased (within the low and medium ranges), the b* value increased very rapidly (Figure 4C). The changes in the b* depended on the copper concentration and the TA contents (Figure 1C). According to previous reports [23,24,25,26], in a model wine system, copper promotes the formation of xanthylium cation pigments, which usually requires tartaric acid and CAT. The xanthylium cation pigments have an absorbance maximum of 440 nm in the visible region, which is similar to the absorbance measured by the wine industry to indicate the browning of wine (i.e., 420 nm) [25]. In this study, the b* value increased with the copper concentration, indicating that the wine color changed to yellow and, thus, this phenomenon also occurs in real wines. Moreover, this color change also indicated that the increasing of TA content (Figure 1C) was primarily due to the changes in the formation of xanthylium cation pigments. The C* value exhibited similar changes to the L* and a* values (Figure 1D), whereas the H* value exhibited similar changes to the b* value (Figure 1E). These results were consistent with a previous report [31]. When copper pollution reached the high copper ranges, all five CIELAB color parameters decreased, because of copper casse formation and the subsequent formation of haze [5,24]. Remarkably, the H* values of the control group and the 96 mg/L group were similar.

Based on the correlation coefficients (Table 3), low copper concentrations increased all color parameters. L*, a*, and C* were negatively correlated and b* and H* were positively correlated with medium copper concentrations. Furthermore, L*, a*, b*, and C* were negatively correlated and H* was positively correlated with high copper concentrations.

### 2.5. Changes in the Antioxidant Activities of Wines with Different Copper Concentrations

Wines have always been considered to have positive effects on human health, mainly because of the antioxidant substances they contain and their associated antioxidant activities [33]. Hence, changes in the antioxidant activities of wines with different copper concentrations were detected in this study. The methods that are usually used to determine the antioxidant activity of wine are assessing free radical scavenging activity, chelating transition metals to prevent the generation of free radicals, and determining the reducing power of samples [18,31]. As a unified standard method for studying antioxidant activity is not available, two or more methods based on different mechanisms are usually chosen to simultaneously explain the antioxidant activity of samples [31,33]. Four different methods, 2,2-diphenyl-1-picrylhydrazyl (DPPH), 2,2’-azino-bis-(3-ethylbenzothiazoline-6-sulfonic acid) diammonium salt (ABTS), oxygen radical absorbance capacity (ORAC), and fluorescence recovery after photobleaching (FRAP), were used in this study.

Since some other elements besides copper, such as iron, zinc, manganese, and cadmium could also influence the phenolic compound contents, color and antioxidant activity of wine [24,34]. Hence, firstly, the contents of iron, zinc, manganese, and cadmium in initial juice and in finished wine were also detected. As shown in Table 2, the contents of iron, zinc, manganese, and cadmium were 6.78 mg/L, 1.78 mg/L, 2.28 mg/L, and 0.75 μg/L in initial juice, respectively. These values were similar with previous reports which using grapes from the same region [35,36]. After fermentation, all these four elements decreased, which was in conformity with the report of Gómez et al. [37], but different from the Cheng et al. report [36], in which the iron and manganese were increased after fermentation. This difference might due to the different crushing process, which using hands and glass vessel in this study and using metal destemmer and transfer tube in the Cheng et al. report. Though all four of these elements decreased after fermentation, in the finished wine of the tested ten copper treated groups in this study, the contents of these four elements showed no significant difference. Hence, the changes of antioxidant activities of wines in this study were mainly due to the different copper treatments, whether it was the residual copper in wines or the changes in the phenolic compound contents caused by copper.

Except copper and other redox active metals, when considering the antioxidant activities of wines, the influence of SO_2_ must be taken into account. SO_2_ was the most important and widely-used chemical in winemaking, which had antimicrobial properties, antioxidant activities, and could prevent wine from browning [23,24,34]. In this study, the SO_2_ contents in finished wine are shown in Table S1. It could be observed that the residual SO_2_ was very low in finished wines. This was because the whole dosage of SO_2_ in this experiment was low. Hence, the influence of SO_2_ on antioxidant activities in this study was tiny. However, in the industrial production, the dosage of SO_2_ was much higher than the present study. Hence, in the future, research needs to be conducted on the influence of copper on wine under industrial production with high dosages of SO_2_.

**Figure 5:**
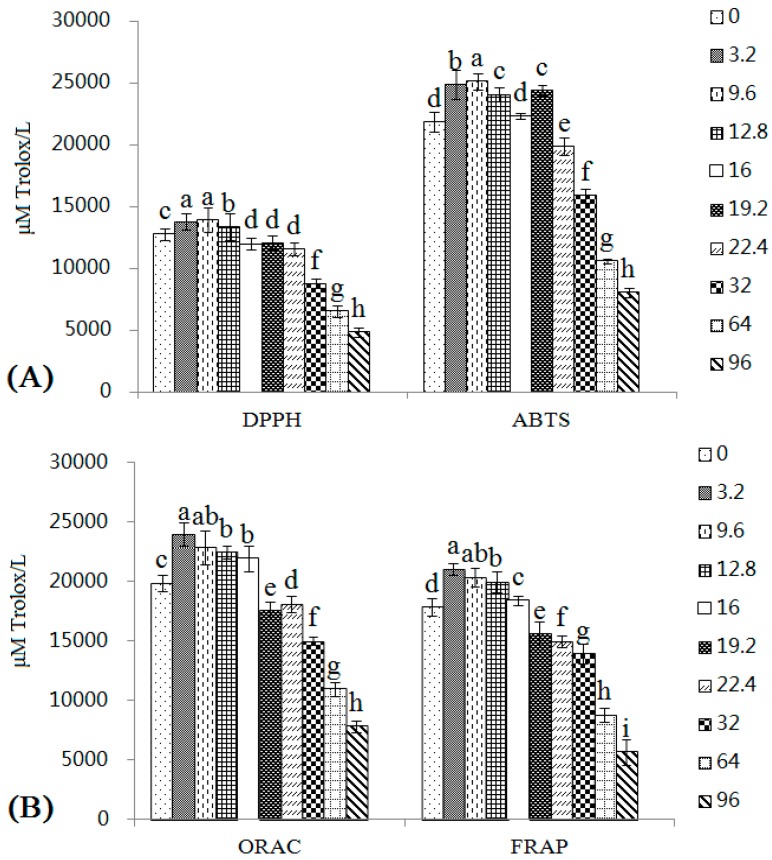
Changes in the antioxidant activities of wines with different copper concentrations were determined using the DPPH radical scavenging activity (μM Trolox/L), ABTS radical scavenging activity (μM Trolox/L) (**A**), ORAC radical scavenging activity (μM Trolox/L) and FRAP radical scavenging activity (μM Trolox/L) methods (**B**). Different letters indicate statistically significant differences (Duncan’s multiple range test, *p* < 0.05) between samples with different copper concentrations.

As shown in Figure 5, the ABTS value was higher than the DPPH value, mainly due to differences between the methods [33]. The values of the four methods all increased in samples with low copper concentrations. At 9.6–16 mg/L, the four methods all showed values that were greater than, or similar to, the control group. In the 19.2 and 22.4 mg/L groups, DPPH, ORAC, and FRAP (but not ABTS) all showed significantly lower values than the control group. When the copper concentrations reached the high pollution range, the antioxidant activities measured by the four methods were all substantially decreased. In fact, in the 96 mg/L group, the DPPH, ABTS, ORAC, and FRAP values were only 38.04%, 36.91%, 81.62%, and 50.96% of the values of the control group.

As shown in Table 4, low copper levels increased the antioxidant activity of wines. Although the antioxidant activity of wines increased in samples containing copper concentrations at the lower end of the medium copper range (9.6–16 mg/L), the antioxidant activity was negatively correlated with all copper concentrations. At high copper concentrations, the antioxidant activity of wines decreased in a dose-dependent manner, one of the main reasons was the changes of the phenolic compound contents in wines, which are the main contributors to the antioxidant activity of wine [18]. The high residual copper in finished wines could be another main reason for the decreasing of antioxidant activity of wines, as copper could also interfere with antioxidant assays [38].

Based on Table 2, though the changes of antioxidant activities of wines in this study were mainly due to the different copper treatments, there still exist many other redox active metals, such as iron, zinc, manganese, and cadmium. In the future, research needs to be conducted in using model wine systems which could completely rule out the influence of other redox active metals, so as to understand the influence of copper on the antioxidant activities of wines more clearly.

To better understand the role of copper on the antioxidant activity, the phenolic compound contents, and the color of wine, the present study is still not sufficient. Further study is still needed, especially a systematic study using the fermentation kinetics to analyze the variable coefficients among copper and the relative parameters, including TP, TFO, TFA, and TA contents, monomeric phenolic acid, flavan-3-ol, and anthocyanin contents, color parameters, and also antioxidant activity values.

## 3. Experimental Section

### 3.1. Materials

Cabernet Sauvignon grapes were donated by the “Baihuagu” winery, which is located in the Huaizhuo basin region of China, in 2015. The original copper concentration was 0.22 mg/L. The *S. cerevisiae* strain used in this study was LALVIN CY3079 (Lallemand, Birkerød, Denmark). All standards were obtained from Sigma-Aldrich (St. Louis, MO, USA), including five flavan-3-ol standards ((+)-catechin (CAT), (−)-epicatechin (EC), (−)-epigallocatechin (EGC), (−)-epicatechin gallate (ECG), and (−)-epigallocatechin gallate (EGCG)) and eleven phenolic standards, including six hydroxybenzoic acids (gallic acid, protocatechuic acid, *p-*hydroxy benzoic acid, gentisic acid, vanillic acid, and syringic acid) and five hydroxycinnamic acids (caffeic acid, *p-*coumaric acid, ferulic acid, chlorogenic acid, and sinapic acid). Folin-Ciocalteu phenol reagent, 2,2-diphenyl-1-picrylhydrazyl (DPPH), 2,2’-azino-bis-(3-ethylbenzothiazoline-6-sulfonic acid) diammonium salt (ABTS), 6-hydroxy-2,5,7,8-tetramethylchroman-2-carboxylic acid (Trolox) and *p-*dimethyl-aminocinnamaldehyde (DMACA) were obtained from Sigma-Aldrich (St. Louis, MO, USA). Deionized water was obtained from Wahaha Co. (Hangzhou, China). Methanol and acetonitrile were of High Performance Liquid Chromatography (HPLC) grade (Spectrum Chemical Co., Irvine, CA, USA), and all of the remaining reagents were of analytical grade.

### 3.2. Fermentation Experiments

The wine vinification process was performed according to the procedure described in previous reports [39,40]. After harvesting, the Cabernet Sauvignon grapes (400 kg) were immediately destemmed and softly crushed using hands, and sulfur dioxide was added (sulfited) to a final concentration of 20 mg/L. The initial Brix was 22.7. Then, the grape must was poured into sterile 10 L glass jars. The grape must was inoculated with 0.25 g/L active dry yeast. A graded series of three different copper content ranges (Table 2) were set by adding CuSO_4_·5H_2_O into the grape must (the original copper concentration was 0.22 mg/L). The fermentation temperature was controlled at 20–25 °C. The cap was punched twice a day during the first few days of fermentation. When the liquid density of the red wines stabilized, the wine and the pomace were separated using two layers of aseptic gauze. The clarified red wines underwent secondary fermentation at 12 °C for six days. The supernatant was then separated and stored at 4 °C. Fermentation was performed in three parallel sets for each treatment.

### 3.3. Measurements of the Total Phenolic (TP), Total Flavonoid (TFA), Total Flavan-3-ol (TFO), and Total Anthocyanin (TA) Concentrations

The TP content was determined using the Folin-Ciocalteu colorimetric method using the calibration curve method [41]. The results are expressed as mg gallic acid equivalents (GAE)/L (mg GAE/L). The TFA content was determined using a previously-described protocol using the calibration curve method [18,42]. The results are expressed as mg catechin equivalents (CTE)/L (mg CTE/L). The TFO content was estimated using a slightly modified DMACA method using the calibration curve method [43]. The results are expressed as mg CTE/L. The TA content was estimated using the pH differential method [33,44]. The results are expressed as mg cyanidin-3-glucoside (CGE)/L (mg CGE/L).

### 3.4. Detection of Phenolic Acids and Flavan-3-ols

A Waters Alliance 2695 HPLC system with a Waters 2996 PDA (photo-diode array) Detector (Waters Corp., Milford, MA, USA) was used to simultaneously separate and analyze the phenolic acids. The system was run at 1.0 mL/min using a LiChrospher 100RP-18e column (250 mm × 4.0 mm) from Merck (Darmstadt, Germany) and an RP-18 guard column (10 mm × 4 mm) from Merck. The column temperature and the injection volume were set at 30 °C and 10 μL, respectively. The detection wavelengths were 280 nm and 320 nm. External standards were used to detect the target analytes. Mobile phase A was water, methanol, and acetic acid (88:10:2), whereas mobile phase B was methanol, water, and acetic acid (90:8:2). The following gradient elution profile was used: 0 to 25 min, mobile phase A from 100 to 85%; 25 to 45 min, mobile phase A from 85 to 50%; and 45 to 53 min, mobile phase B from 50 to 100%. All samples were filtered through a 0.45 μm Poly Tetra Fluoro Ethylene (PTFE) Millipore filter before injection (Merck Millipore, Billerica, MA, USA) [17,21]. The quantitative method was conducted using the calibration curve method. The mixed standard of 11 phenolic acids was formulated into a solution in chromatography-grade methanol. The liquid chromatogram is shown in Figure S2A. It can be seen that good separation is achieved for the 11 phenolic acids under the chromatographic conditions, as well as a good limit of detection (Table S2).

A Waters Alliance 2695 HPLC system with a Waters 2996 PDA Detector was also used to simultaneously separate and analyze flavan-3-ols. The system was run at 1.0 mL/min, with the same chromatographic column and guard column used to detect the phenolic acids. The wavelength was 280 nm, the column temperature was 30 °C, and the injection volume was 10 μL. External standards were used to detect the target analyte. Mobile phase A was water. Mobile phase B was acetic acid and water (10:90). The following gradient elution profile was used: 0 to 20 min, mobile phase A from 92.5 to 35%; 20 to 30 min, mobile phase A from 35 to 20%; 30 to 48 min, mobile phase A from 20 to 10%; 48 to 55 min, mobile phase A at 10%; and 55 to 63 min, mobile phase A from 10 to 92.5%. Samples were filtered through a 0.45 μm poly tetra fluoro ethylene (PTFE) Millipore filter before injection [17,21]. The quantitative method used the calibration curve method. The mixed standard of five flavan-3-ols was formulated into a solution in chromatography-grade methanol. The liquid chromatogram is shown in Figure S2B. It could be seen that good separation is achieved for the five flavan-3-ols under the chromatographic conditions as well as a good limit of detection (Table S3).

### 3.5 Color Parameter Measurements

The color parameters were measured using the CIELAB space, according to a previously-described method [30,31]. The samples were filtered through 0.45-μm filter membranes. The measurements were performed in a Shimadzu UV-1800 spectrophotometer (Kyoto, Japan) using a 0.2 cm path-length quartz cuvette. Absorbance was measured at 450, 520, 570, and 630 nm using distilled water as the blank control. L*, a*, and b* were calculated according to the following formulas:

τ = 10^−A^,

X = 19.717τ_450_ + 1.884τ_520_ + 42.539τ_570_ + 32.474τ_630_ − 1.841,

Y = 7.950τ_450_ + 34.764τ_520_ + 42.736τ_570_ + 15.759τ_630_ − 1.180,

Z = 103.518τ_450_ + 4.190τ_520_ + 0.251τ_570_ – 1.831τ_630_ + 0.818,

L* = 116((Y/Y_10_)^1/3^ − 0.1379), a* = 500((X/X_10_)^1/3^ − (Y/Y_10_)^1/3^

b* = 200((Y/Y_10_)^1/3^ − (Z/Z_10_)^1/3^)

where A represents the absorbance, τ represents transmittance, and the tristimulus values for the blank with a D65 illuminant and CIE1964 standard observer are X_10_ = 94.825, Y_10_ = 100, and Z_10_ = 107.381. The H* (H* = arctan b*/a*) and C* (C* = [(a*)^2^ + (b*)^2^]^0.5^) were also calculated [30].

### 3.6. Analysis of Antioxidant Capacity

Four different methods, DPPH, ABTS, ORAC and FRAP, were used in this study. The DPPH scavenging activity and ABTS assays were based on previously-described methods [45,46,47], with slight modifications. The oxygen radical absorbance capacity (ORAC) assay and fluorescence recovery after photobleaching (FRAP) assays were performed essentially as has been previously described [48,49], with some modifications. The results are expressed as μM Trolox/L.

### 3.7. Analysis of Copper, Iron, Zinc, Manganese, and Cadmium

#### 3.7.1. Sample Pretreatment

For grape must, 3 mL of grape must was placed into a 50-mL conical flask and baked in a dust-free oven at 105 °C until sticky. Following baking, 5 mL HNO_3_ (65%)-HClO_4_ (70%) (4:1, guaranteed reagent) was mixed into the conical flask, which was then covered with a lid and heated on an electric hot plate at 80 °C for 2 h, 120 °C for 2 h, and then 190 °C, until the mixture no longer emitted white smoke and the solution was colorless or light yellow. After this, the mixture was cooled to ambient temperature and diluted to 25 mL with ultrapure water for further analyses. All glassware was soaked overnight with 20% HNO_3_, washed with ultrapure water, and dried prior to use [5,6].

For wine samples, each sample was diluted 10 times using 5% nitric acid (guaranteed reagent) before further analyses [13,50].

#### 3.7.2. ICP-OES Determination

The copper, iron, zinc, manganese, and cadmium contents were detected using an inductively-coupled plasma optical emission spectrometer (ICP-OES, Optima 7000DV, Perkin Elmer) method [5,6,50]. Diluted copper, iron, zinc, manganese, and cadmium standard stock solutions at different concentrations, using 5% HNO_3_, were used to draw the standard curves. Before injection, all samples were filtered with a 0.45 µm water membrane.

### 3.8 Analysis of SO_2_

The SO_2_ content was determined using National Standard of China GB/T 15038-2006 [51].

### 3.9. Statistical Analysis

The experimental results are expressed as the means ± standard deviations (SD) of three replicate fermentations for each treatment. Correlations were calculated using a linear regression. Statistical analyses were performed using Data Processing System software (DPS, version 7.05, Hangzhou, China) [52].

## 4. Conclusions

In summary, copper had a clear effect on wine polyphenol content. At low copper concentrations, the contents of nearly all phenolic compounds increased, and the color parameter values and antioxidant activity values of the wine also increased. When the copper concentrations reached the lower range of the medium copper concentrations (9.6–16 mg/L), most of the indices were improved. When the copper concentrations reached the upper range of the medium copper concentrations (19.2 and 22.4 mg/L, which was near, or slightly greater than, the legal limits of the EU and OIV), many of the tested indices began to decrease. Moreover, when the copper concentrations reached the high range (32, 64, and 96 mg/L, which might occur in the future), the phenolic compound contents, CIELAB color parameters, and antioxidant activity of wine were all substantially decreased, indicating the need to control the increasing copper content in grape must. Specifically, the TA, EGC, and ECG contents, and b* values, were substantially increased in samples with low and medium copper concentrations, whereas the CAT levels were significantly decreased. These phenomena may be correlated, but further studies are required to confirm this hypothesis.

## Figures and Tables

**Table 1:** Lineal correlation coefficients (R^2^) between copper concentrations and phenolic compounds of wine.

Phenolic Compounds	Copper Concentrations			Phenolic Compounds	Copper Concentrations		
**Hydroxybenzoic Acids**	**Low**	**Medium**	**High**	**Flavonols**	**Low**	**Medium**	**High**
gall	+	−0.5468	−0.9155	CAT	+	−0.8743	−0.7586
prot	+	−0.3135	−0.9757	EGC	+	+0.0977	−0.9960
*p-*hyd	+	+0.0747	−0.8834	EGCG	+	+0.6165	−0.9540
gent	+	−0.0081	−0.9757	EC	+	−0.0032	−0.8421
vani	+	−0.1141	−0.9378	ECG	+	+0.9001	−0.9918
syri	+	−0.0014	−0.9844	*TMFA*	+	−0.6257	−0.9579
THBA	+	−0.4473	−0.9565				
**Hydroxycinnamic Acid**							
caff	+	+0.1286	−0.9748	TP	+	−0.7783	−0.9995
*p-*cou	+	+0.0071	−0.9933	TFA	+	−0.6335	−0.8103
feru	+	+0.0005	−0.9764	TFO	+	−0.8351	−0.9333
sina	−	−0.4541	−0.9124	TA	+	+0.6744	−0.8596
chlo	+	+0.7274	−0.9227				
THCA	+	+0.0265	−0.9712				
TPA	+	−0.3775	−0.9774				

Note: Values expressed as Pearson correlation coefficient (r).

**Table 2:** Contents of copper, iron, zinc, manganese and cadmium in initial juice and in finished wine.

Sample		Element				
		Copper	Iron	Zinc	Manganese	Cadmium
Initial juice		0.22 ± 0.02 d	6.78 ± 0.21 a	1.78 ± 0.09 a	2.28 ± 0.09 a	0.75 ± 0.04 a
Finished wine						
Low	0.00 mM (0 mg/L)	0.04 ± 0.00 a	5.66 ± 0.43 b	1.44 ± 0.14 b	1.98 ± 0.19 b	0.56 ± 0.05 b
	0.05 mM (3.2 mg/L)	0.05 ± 0.01 a	5.71 ± 0.35 b	1.35 ± 0.11 b	1.87 ± 0.22 b	0.65 ± 0.05 b
Medium	0.15 mM (9.6 mg/L)	0.06 ± 0.01 a	5.38 ± 0.47 b	1.52 ± 0.15 b	1.79 ± 0.17 b	0.58 ± 0.07 b
	0.20 mM (12.8 mg/L)	0.07 ± 0.02 a	5.74 ± 0.61 b	1.39 ± 0.19 b	1.85 ± 0.14 b	0.49 ± 0.04 b
	0.25 mM (16.0 mg/L)	0.11 ± 0.02 b	5.41 ± 0.41 b	1.45 ± 0.09 b	1.91 ± 0.21 b	0.55 ± 0.08 b
	0.30 mM (19.2 mg/L)	0.15 ± 0.03 bc	5.32 ± 0.34 b	1.51 ± 0.12 b	1.78 ± 0.24 b	0.59 ± 0.05 b
	0.35 mM (22.4mg/L)	0.16 ± 0.02 c	5.56 ± 0.55 b	1.45 ± 0.14 b	1.89 ± 0.18 b	0.61 ± 0.04 b
High	0.50 mM (32 mg/L)	2.18 ± 0.19 e	5.78 ± 0.37 b	1.56 ± 0.11 b	1.99 ± 0.18 b	0.63 ± 0.06 b
	1.00 mM (64 mg/L)	4.67 ± 0.23 f	5.89 ± 0.44 b	1.58 ± 0.09 b	2.01 ± 0.25 b	0.64 ± 0.04 b
	1.50 mM (96 mg/L)	7.59 ± 0.44 g	5.97 ± 0.41 b	1.55 ± 0.12 b	2.09 ± 0.18 b	0.67 ± 0.05 b

Note: the units for copper, iron, zinc and manganese were mg/L, while for cadmium was μg/L. And different letters (a, b, c) in each of the columns indicate that the values are significantly different (*p* < 0.05).

**Table 3:** Lineal correlation coefficients (R^2^) between copper concentrations and color of wine.

Color Parameters	Copper Concentrations		
	Low	Medium	High
L*	+	−0.1107	−0.9129
a*	+	−0.3869	−0.8722
b*	+	+0.8639	−0.9686
C*	+	−0.0839	−0.8791
H*	+	+0.8454	+0.3778

**Table 4:** Lineal correlation coefficients (R^2^) between copper concentrations and antioxidant activity of wine.

Antioxidant Activity	Copper Concentrations		
	Low	Medium	High
DPPH	+	−0.4678	−0.9952
ABTS	+	−0.1099	−0.9600
ORAC	+	−0.2933	−0.9953
FRAP	+	−0.4330	−0.9804

## Supplementary Materials

The Supplementary Materials are available online.
