# Supplementary material for: Effects of Copper Pollution on the Phenolic Compound Content, Color, and Antioxidant Activity of Wine
Source: Molecules. 2017 May 3;22(5):726. doi: 10.3390/molecules22050726 (PMC6154001; doi:10.3390/molecules22050726)
Supplement: Supplementary file 1 [file molecules-22-00726-s001.pdf]

Supplementary Materials: Effects of Copper Pollution on the Phenolic Compound Content, Color, and Antioxidant Activity of Wine

Xiangyu Sun, Tingting Ma, Luyang Han, Weidong Huang and Jicheng Zhan

Table S1. Sulfur dioxide (SO_2) contents in initial juice and in finished wine.

Sample		SO_2 Content (mg/L)
Initial juice		20 ± 0.00 a
Finished wine		
Low	*0.00 mM (0 mg/L)*	1.01 ± 0.11 b
	0.05 mM (3.2 mg/L)	1.05 ± 0.06 b
Medium	*0.15 mM (9.6 mg/L)*	1.06 ± 0.11 b
	0.20 mM (12.8 mg/L)	1.07 ± 0.12 b
	0.25 mM (16.0 mg/L)	1.11 ± 0.12 b
	0.30 mM (19.2 mg/L)	1.09 ± 0.13 b
	0.35 mM (22.4 mg/L)	1.06 ± 0.12 b
High	*0.50 mM (32 mg/L)*	0.98 ± 0.09 b
	1.00 mM (64 mg/L)	1.01 ± 0.03 b
	1.50 mM (96 mg/L)	0.92 ± 0.14 b

Table S2. Detection wavelength, retention time, regression equation and limit of detection of eleven phenolic acid compounds.

Phenolic Compounds	Detection Wavelength λ/nm	Retention Time t/min	Linear Range w/(mg/L)	Regression Equation	R^2	Limit of Detection ϱ/(mg/L)
gallic acid	280	4.904	1–150	y = 65,231x − 18,205	0.9995	0.0252
protocatechuic acid	280	9.332	1.4–140	y = 73,473x − 32,526	0.9993	0.6546
gentisic acid	320	13.743	1.5–100	y = 152,346x − 76,574	0.9998	0.2145
p-hydroxy benzoic acid	280	15.788	0.5–100	y = 36,453x − 13,256	0.9998	0.0643
chlorogenic acid	280	20.102	0.5–100	y = 274,682x − 76,543	0.9999	0.0155
vanillic acid	280	21.508	1–100	y = 154,654x − 63,263	0.9992	0.0145
caffeic acid	280	22.656	1–100	y = 117,543x − 16,473	0.9997	0.2261
syringic acid	280	26.358	0.5–50	y = 86,234x − 65,546	0.9998	0.0462
p-coumaric acid	280	33.232	0.5–50	y = 153,435x − 34,511	0.9998	0.0154
ferulic acid	280	36.714	0.5–50	y = 765,425x − 65,342	0.9995	0.0233
sinapic acid	280	37.792	0.5–50	y = 91,543x − 65,432	0.9997	0.0235

Table S3. Detection wavelength, retention time, regression equation and limit of detection of five flavan-3-ol compounds.

Phenolic Compounds	Detection Wavelength λ/nm	Retention Time t/min	Linear Range w/(mg/L)	Regression Equation	R^2	Limit of Detection ϱ/(mg/L)
(+)-Catechin	280	22.866	1–100	y = 124,945x − 16,335	0.9993	0.0726
(−)-Epigallocatechin	280	23.823	1–100	y = 163,433x − 42,695	0.9998	0.0423
EGCG	280	31.611	1–100	y = 154,366x − 43,266	0.9998	0.1355
(−)-Epicatechin	280	35.925	1–100	y = 436,241x − 15,345	0.9999	0.0255
(−)-Epicatechin gallate	280	50.913	1–100	y = 515,634x − 43,624	0.9997	0.0632

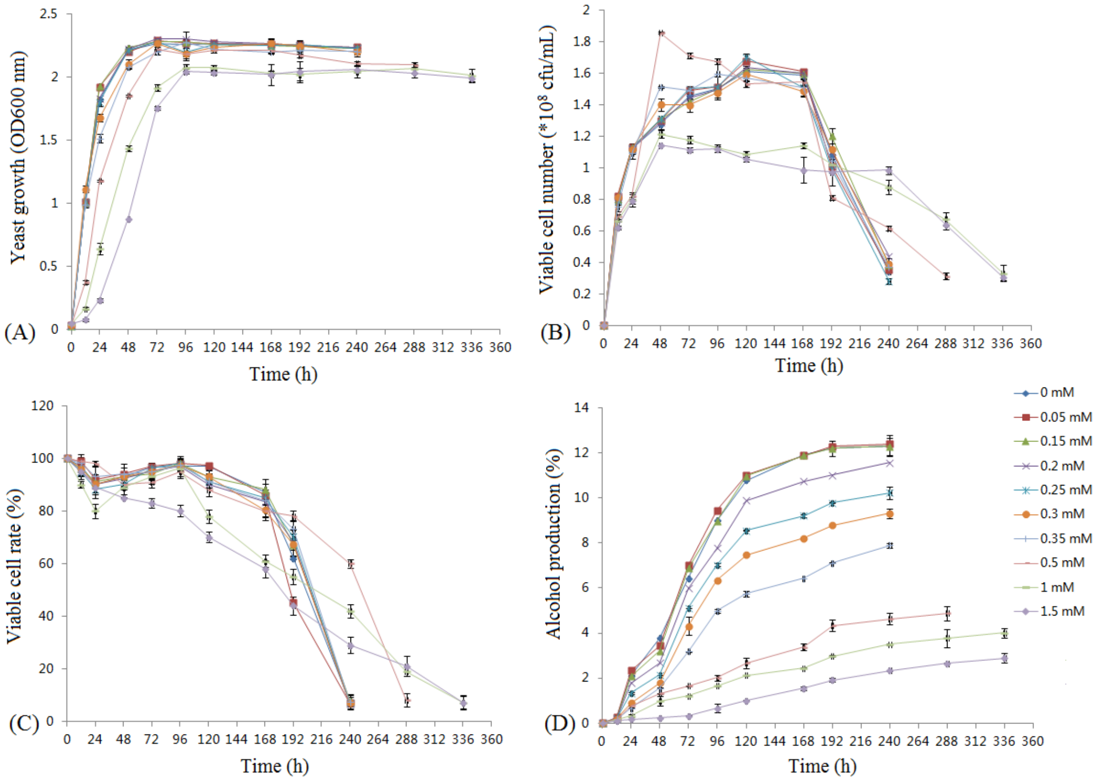

Figure S1. Yeast growth (**A**), viable cell number (**B**), viable cell rate (**C**), and alcohol production (**D**).

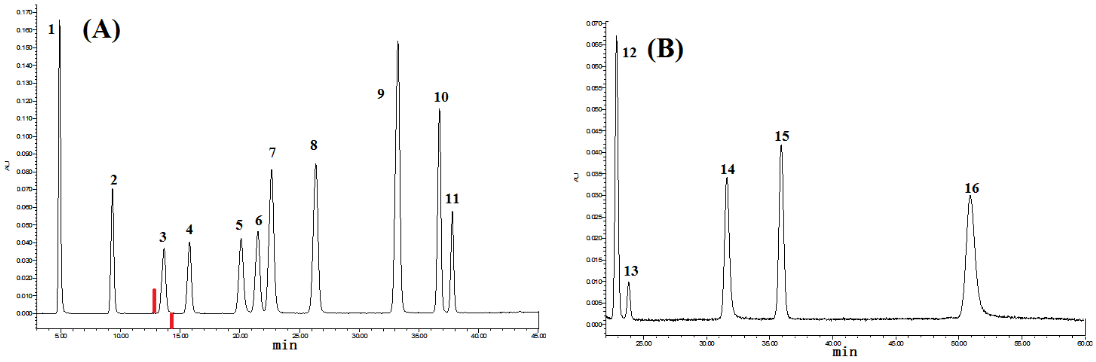

Figure S2. HPLC chromatographs of 11 phenolic acids (**A**) (gentisic acid was detected at 320 nm (the red markers), the others at 280 nm) and (**B**) 5 flavan-3-ol standards (280 nm). 1, gallic acid; 2, protocatechuic acid; 3, gentisic acid (320 nm); 4, *p*-hydroxybenzoic acid; 5, chlorogenic acid; 6, vanillic acid; 7, caffeic acid; 8, syringic acid; 9, *p*-coumaric acid; 10, ferulic acid; 11, sinapic acid; 12, (+)-catechin; 13, (−)-epigallocatechin; 14, (−)-epigallocatechin gallate; 15, (−)-epicatechin and 16, (−)-epicatechin gallate.
